# The Human Knee: Gross, Microscopic, Surgical, and Radiological Anatomy

**DOI:** 10.1155/2012/698346

**Published:** 2012-12-10

**Authors:** Konstantinos Natsis, Nikolaos Anastasopoulos, Eleftherios Kellis, Juergen Koebke, Antonia Sioga, Ioannis Tsitouridis

**Affiliations:** ^1^Department of Anatomy, Medical School, Aristotle University of Thessaloniki, 54124 Thessaloniki, Greece; ^2^Department of Physical Education and Sport Sciences, Aristotle University of Thessaloniki, 62110 Serres, Greece; ^3^Department of Anatomy, University of Cologne, 50931 Cologne, Germany; ^4^Department of Histology and Embryology, Medical School, Aristotle University of Thessaloniki, 54124 Thessaloniki, Greece; ^5^Department of Radiology, General Hospital Papageorgiou, 56429 Thessaloniki, Greece

During the last years imaging techniques have rapidly developed in anatomy since they were included in the study of the human body. Normal patterns have been revisited and lately, in the era of evidence-based medicine, anatomy has shifted towards evidence-based morphology. Endoscopic and minimally invasive techniques require a different view and a better understanding of anatomy. Individualized patient care requires understanding of the individualized anatomy especially with regard to surgery. Anatomy is more well timed than ever and the papers selected for this special issue reflect the modern era of anatomy. The papers included are descriptive studies aiming to describe anatomy but they somehow represent different study designs and all of them have been conducted under a clinically orientated perspective. Although the human knee has been extensively studied, it seems that there are still research questions that need to be addressed. We would like to thank the authors for their contributions to this special issue. The fundamental work of all the reviewers is also acknowledged.

In the paper “*The ‘oblique popliteal ligament:' a macro- and microanalysis to determine if it is a ligament or a tendon*” B. Benninger and T. Delamarter challenge a well-known structure, the oblique popliteal ligament (OPL). Based on their observations the authors suggest that the OPL is indigenous to the distal semimembranosus muscle tendon unit. The microanalysis using an immunohistochemistry stain with PGP9.5 revealed a positive result for neuronal axons within both the semimembranosus tendon and OPL. Further microanalysis using an immunohistochemistry stain with **β**-tubulin revealed a positive stain for neuronal axons in the semimembranosus tendon, OPL, and lateral collateral ligament. Though the latter result leads the authors to question the validity of differentiating the tendon from ligament using this particular immunohistochemistry stain, the macroanalysis results are overwhelming, and the microanalysis reveals striking similarities in the histology of both the OPL and semimembranosus tendon.

In the paper entitled “*The patellar arterial supply via the infrapatellar fat pad (of Hoffa): a combined anatomical and angiographical analysis*” G. Nemschak and M. L. Pretterklieber describe the rich patellar arterial supply provided via the infrapatellar fat pad (of Hoffa). Five human patellae, one was dissected under the operation microscope, a second was made translucent by Sihlers-solution, and three underwent angiography using a 3D X-ray unit, were studied. The results revealed that the patella to a considerable amount is supplied by arteries coursing through the surrounding parts of the infrapatellar fat pad. The latter were found to branch off from the medial and lateral superior and inferior genicular arteries. Within the infrapatellar fat pad, these arteries formed a dense network of anastomoses which are all contributing to the viability of the patellar bone. The authors conclude that due to the rich arterial supply reaching the patella via the infrapatellar fat pad, it seems advisable to preserve the fat pad during the surgery of the knee in order to reduce the risk of vascular impairment of the patella.

In the paper “*Meniscofibular ligament: morphology and functional significance of a relatively unknown anatomical structure*” K. Natsis et al. performed a gross and microscopic study on the meniscofibular ligament (MFL). Based on their observations the authors speculated that MFL could offer protection to the lateral meniscus from likely damage during the last stages of knee extension. Moreover, MFL seems to reinforce the posterolateral part of the lateral coronary ligament, a fact that could explain the relative low incidence of lateral coronary ligament rupture. This study formulated research questions for further research with regard to the exact biomechanical characteristics of the MFL, as well as the likely relation or not to lateral meniscus tears, protection of the coronary ligament, and function and traumatology of the proximal tibiofibular joint.

In the paper “*Application of soft tissue artifact compensation using displacement dependency between anatomical landmarks and skin markers*” T. Ryu presents a different view of anatomy applications with regard to joint analysis. Among many approaches to reduce errors in motion analysis, by means of stereophotogrammetry, is to estimate the position of anatomical landmarks during a motion with joint angle or displacement of skin markers, which is the so-called compensation method of anatomical landmarks. This study aimed to apply the compensation methods with joint angle and skin marker displacement to three lower extremity motions and to compare their reliability. Two sets of kinematic variables were calculated using two different marker clusters, and the difference was obtained. Results showed that the compensation method with skin marker displacement had less differences by 30–60% compared to without compensation. In addition, it had significantly less differences in some kinematic variables (7 of 18) by 25–40% compared to the compensation method with joint angle.

In the paper entitled “*Adequacy of semitendinosus tendon alone for anterior cruciate ligament reconstruction graft and prediction of hamstring graft size by evaluating simple anthropometric parameters*” Papastergiou G. S. et al. performed a retrospective study of a clinical population in order to determine whether the semitendinosus tendon length is adequate for a four-strand graft harvested by common technique and if there is a correlation of gracilis and semitendinosus grafts length and diameter with anthropometric parameters. According to the findings of the study the authors concluded that the length of semitendinosus tendon, harvested by the common technique, is usually inadequate in order to be used alone as a four-strand graft especially in females. Height and weight are considered to be moderate predictors of the adequacy of the semitendinosus tendon length when using alone the semitendinosus four-strand graft or of the four-strand semitendinosus and gracilis graft diameter for anterior cruciate ligament single-bundle reconstruction harvested by common technique (without bone plug). The most reliable predictor seems to be patients' height in males. In female patients, there is no such statistically important predictor.

In the paper “*Gender and side-to-side differences of femoral condyles morphology: osteometric data from 360 caucasian dried femori*” I. Terzidis et al. performed a population-based morphometric study of the femoral condyles.

It is still a controversial issue whether a new implant design might rather take into account interindividual variations in the knee joint anatomy instead of gender-specific variations. The authors highlighted differences in anatomy between genders that might add to the design of new prostheses. Based on the results of this study, the contralateral healthy side can be used safely for preoperative templating in total knee reconstruction surgery since no side-to-side differences were found.

In the paper “*Anterior and posterior meniscofemoral ligaments: MRI evaluation*” A. Bintoudi et al. provide an overview of the MRI appearance of the anterior meniscofemoral and posterior meniscofemoral ligaments. The authors have used a large sample size to describe the imaging anatomy of these structures which aids in better conceptualization of the anatomy and averts misdiagnosis of the anterior meniscofemoral and posterior meniscofemoral ligaments as loose bodies or posterior cruciate ligament pathology.

*Konstantinos Natsis* *Nikolaos Anastasopoulos* *Eleftherios Kellis* *Juergen Koebke* *Antonia Sioga* *Ioannis Tsitouridis*

